# ^18^F-FDG PET/CT in the evaluation of femoral head osteonecrosis in patients with lymphoma

**DOI:** 10.1186/s12880-026-02520-y

**Published:** 2026-06-25

**Authors:** Le Song, Hui Li, Anhui Zhu, Weifang Zhang

**Affiliations:** Department of Nuclear Medicine, Peking University Third Hospital, 49 North Garden Rd, Haidian District, Beijing, 100191 China

**Keywords:** Femoral head, Osteonecrosis, Lymphoma, Positron emission tomography-computed tomography, Magnetic resonance imaging

## Abstract

**Objective:**

To describe the imaging features of osteonecrosis of the femoral head (ONFH) on ^18^F-fluorodeoxyglucose (^18^F-FDG) positron emission tomography (PET)/CT and explore whether PET/CT can raise suspicion for ONFH in patients with lymphoma.

**Methods:**

A retrospective analysis was conducted on the clinical data, PET/CT, and MRI findings of 17 patients with lymphoma and ONFH. The ^18^F-FDG uptake of ONFH was recorded, and the maximum standardized uptake value (SUV_max_) of ONFH was measured. The staging and extent of ONFH, and other bone involvements, were visually assessed. Lymphoma disease status was evaluated using the Deauville criteria.

**Results:**

A total of 31 femoral heads were involved (2 stage 1, 24 stage 2, 4 stage 3, 1 stage 4). The median SUV_max_ was higher in stage 3–4 lesions (5.27) than in stage 1–2 lesions (1.37) (*P* = 0.002). Regarding ^18^F-FDG uptake patterns, all stage 1 lesions showed normal uptake; among stage 2 lesions, 11 showed increased uptake, 5 showed peripheral increased with central decreased uptake, 2 showed decreased uptake, and 6 showed normal uptake; all stage 3–4 lesions demonstrated increased uptake. On CT, the extent of all stage 2–4 lesions matched MRI findings. Nine patients had osteonecrosis in other bones, including the humeral heads (*n* = 9), bilateral iliac bones (*n* = 6), and vertebrae/ribs/scapulae (*n* = 1).

**Conclusions:**

In patients with lymphoma, ONFH exhibits variable degrees of ^18^F-FDG uptake and may be accompanied by involvement of other bones. PET/CT may raise suspicion for ONFH and detect multiple bone involvements during lymphoma assessment.

**Supplementary Information:**

The online version contains supplementary material available at https://doi.org/10.1186/s12880-026-02520-y.

## Background

Osteonecrosis of the femoral head (ONFH) is a common disease of the hip joint and characterized by death of the osteocytes and the bone marrow. The main etiological factors for non-traumatic ONFH include the application of corticosteroids, chronic alcohol overconsumption, decompression sickness, haemoglobin diseases, autoimmune diseases, etc [1, 2]. Patients treated for lymphoma are at high risk of developing ONFH, with an incidence rate of approximately 15% [3]. Moreover, osteonecrosis in patients with hematologic malignancies has been reported to involve multiple bones and joints, rather than being confined to the femoral heads [4]. Early diagnosis is essential for optimal therapeutic management. Once the disease progresses to an advanced stage, joint collapse may occur, and surgical intervention is often required [5, 6]. It is recommended that for patients who complain of hip pain during or after the treatment of lymphoma, a hip MRI examination is suggested to diagnose ONFH [3]. Nevertheless, Ratcliffe et al. reported that 40% of patients with ONFH were asymptomatic [3]. Whether asymptomatic patients at risk should be screened remains unclear. Asymptomatic osteonecrosis has a high prevalence (59%) of progression to symptomatic disease and femoral head collapse [7]. It seems impractical to perform hip X-rays or even MRIs for all asymptomatic patients with lymphoma. It is also difficult to determine the timing and frequency of the screening examinations.

^18^F-fluorodeoxyglucose (^18^F-FDG) positron emission tomography (PET)/CT has been an important imaging modality for the staging and therapeutic evaluation of lymphoma [8, 9]. We speculate that ^18^F-FDG PET/CT also has a role in the evaluation of ONFH for patients with lymphoma. Studies about the value of ^18^F-FDG PET/CT for osteonecrosis are limited to a few case reports [10–13]. This retrospective study aims to describe the imaging features of ONFH on ^18^F-FDG PET/CT and explore whether PET/CT can raise suspicion for ONFH in patients with lymphoma.

## Methods

### Study population

This was a retrospective study and was approved by the institutional review board of Peking University Third Hospital. The PET/CT image database was searched with the keywords “femoral head” and “osteonecrosis” in patients with lymphoma. All of the retrieved patients underwent magnetic resonance imaging (MRI) scan of the hip and were diagnosed as ONFH. Patients with previously known ONFH before the diagnosis of lymphoma or incomplete clinical data were excluded. A total of 17 patients with ONFH were recruited at Peking University Third Hospital between September 2014 and June 2024. We recorded the symptoms such as aches around the hip joint or at the buttocks. No control group of lymphoma patients without ONFH was included.

### PET/CT examination

A Siemens Biograph 64 PET/CT scanner was applied for image acquisition. ^18^F-FDG was provided by Beijing Atomic High Technology Co. Patients fasted for longer than 6 h, and blood glucose levels were monitored (below 11.1 mmol/L) before an injection of ^18^F-FDG (5.55 MBq per kilogram of body weight). About 60 min after injection, the PET/CT was performed from the tip of the skull to the mid-thigh. CT scan parameters: tube voltage 120 kV, effective tube current 100 mAs, pitch 0.9. PET scans were acquired over the same extent as CT, with an acquisition time of 2 min per bed, True X reconstruction, 3 iterations, 21 subsets, Gaussian filtering, and a half-peak width of 5.0.

### MRI examination

MRI of the bilateral hips was performed on 3.0T scanners (GE Medical Systems, Siemens, and United Imaging Healthcare) using the following sequences: axial T1-weighted fast spin-echo (T1 FSE), axial T2-weighted FSE (with or without fat saturation) or axial proton density-weighted FSE with fat saturation (PD FSE FS); coronal T1 FSE, coronal T2 IDEAL (generating in-phase, out-of-phase, fat, and water images) or coronal short tau inversion recovery (STIR). Slice thickness ranged from 3.5 to 4.0 mm. In two patients, MRI was performed for pelvic cavity evaluation rather than dedicated hip imaging; however, the femoral heads were fully included in the field of view, and the essential sequences for diagnosing ONFH were complete.

### Image analysis

The PET/CT images were transferred to the SIEMENS syngoMMWP VE40A workstation. Two nuclear medicine physicians, both with over 10 years of experience in PET/CT, jointly assessed the PET/CT images. In cases where their opinions diverged, a more senior physician was consulted to make the final decision. The readers were not blinded to the MRI results, as the study aimed to describe PET/CT findings in patients with known ONFH. The ONFH was diagnosed and staged according to the modified 2019 version of the Association Research Circulation Osseous international classification of osteonecrosis staging system [1]: stage 1, a band lesion of low signal intensity around the necrotic area is seen on MRI, but no changes are seen on CT; stage 2, osteosclerosis or cystic changes are seen in the femoral head without evidence of subchondral fracture, fracture in the necrotic portion or flattening of the femoral head on CT; stage 3, subchondral fracture, fracture in the necrotic portion and/or flattening of the femoral head on CT; stage 4, osteoarthritis of the hip joint with joint space narrowing, acetabular changes and destruction. The ^18^F-FDG uptake of ONFH was compared with that of the surrounding unaffected femoral head. For the semi-quantitative assessment of ONFH, regions of interest (ROIs) were manually delineated on the PET/CT fused images. The maximum standardized uptake value (SUV_max_) is calculated as ROI activity (mCi/mL) multiplied by body weight (g), divided by the injected dose (mCi). The extent of ONFH on CT was visually evaluated and compared with that on MRI. A lesion-by-lesion comparison was performed for each femoral head, with MRI serving as the reference standard for both staging and extent assessment. Additionally, all other bones covered by the PET/CT scan were evaluated for signs of osteonecrosis. Osteonecrosis of non-femoral sites (e.g., humeral heads, iliac bones) was diagnosed based on the presence of typical CT features (e.g., linear high density, geographic osteosclerosis) and/or corresponding MRI findings, when available. For patients who underwent multiple PET/CT examinations, the ^18^F-FDG metabolism and CT features of ONFH at different time points were analyzed. The disease status of lymphoma was assessed based on the Deauville criteria on the same ^18^F-FDG PET/CT scan used for analysis.

### Statistical Analysis

Statistical analyses were conducted using SPSS Statistics version 26.0 (IBM Corp., Armonk, NY, USA). The Shapiro-Wilk test was employed to assess the normality of data distribution. Data were presented as mean ± standard deviation if they followed a normal distribution; otherwise, they were expressed as median (quartile 1, quartile 3). The Mann-Whitney U test was utilized to compare the SUV_max_ of ONFH at different stages. A *P* value less than 0.05 was considered to indicate statistical significance.

## Results

### Clinical characteristics

A total of 17 patients with ONFH (14 men and 3 women; median age 40 years) were enrolled in this retrospective study. All patients had non-Hodgkin lymphoma. The clinical characteristics, PET/CT findings, and lymphoma disease status (assessed by the Deauville score) are summarized in Table 1. At the time of the MRI examination, 6 patients complained of hip or thigh discomfort, such as intermittent or persistent pain and soreness, while the remaining patients did not report local discomfort.

**Table 1:** Clinical characteristics of enrolled patients and PET/CT findings of osteonecrosis of the femoral head

No.	Gender	Age (years)	Type of lymphoma	Osteonecrosis of the femoral head					Other bone involvement
				Deauville score	Right		Left		
					SUV_max_	ARCO stage	SUV_max_	ARCO stage	
1	Male	43	Diffuse large B-cell lymphoma	1	4.27	2	6.03	2	Bilateral iliac bones, humeral heads
2	Male	40	Diffuse large B-cell lymphoma	1	1.42	2	8.06	3	Left humeral head
3	Male	32	High-grade B-cell lymphoma	2	1.31	2	1.06	2	No
4	Female	25	High-grade B-cell lymphoma	1	1.82	1	2.71	2	Bilateral iliac bones, humeral heads
5	Male	32	T-cell lymphoblastic lymphoma	1	0.88	2	0.98	2	Bilateral iliac bones, humeral heads, ribs, scapulae and vertebrae
6	Male	64	Diffuse large B-cell lymphoma	5	0.55	1	-	-	No
7	Male	25	T-cell lymphoblastic lymphoma	3	1.53	2	2.02	2	Bilateral iliac bones, humeral heads
8	Male	42	Diffuse large B-cell lymphoma	4	1.14	2	1.00	2	No
9	Male	33	T-cell lymphoblastic lymphoma	1	5.45	3	1.30	2	Bilateral iliac bones, humeral heads
10	Male	52	Diffuse large B-cell lymphoma	2	0.72	2	0.85	2	Bilateral humeral heads
11	Male	44	Mantle cell lymphoma	1	4.41	3	2.23	2	No
12	Female	73	High-grade B-cell lymphoma	1	1.65	2	2.12	2	No
13	Female	22	Diffuse large B-cell lymphoma	1	1.59	2	2.64	3	Bilateral iliac bones, humeral heads
14	Male	20	T-cell lymphoblastic lymphoma	1	0.56	2	0.45	2	Bilateral humeral heads
15	Male	67	Mantle cell lymphoma	5	5.27	4	-	-	No
16	Male	27	T-cell lymphoblastic lymphoma	5	1.82	2	1.28	2	No
17	Male	52	Diffuse large B-cell lymphoma	1	1.63	2	-	-	No

*ARCO* Association Research Circulation Osseous, *SUV*_*max*_ maximum standardized uptake value

### PET/CT findings

PET/CT was performed before or after MRI with a median time interval of 3 days (range, 0–98 days). A total of 31 femoral heads were involved (14 bilateral, 3 unilateral), comprising 2 in stage 1, 24 in stage 2, 4 in stage 3, and 1 in stage 4. The median SUV_max_ value was 1.37 (0.96, 1.87) for stage 1–2 lesions and 5.27 (3.53, 6.76) for stage 3–4 lesions. The SUV_max_ of stage 3–4 ONFH lesions was significantly higher than that of stage 1–2 lesions (*P* = 0.002). On maximum intensity projection (MIP) images, increased ^18^F-FDG uptake was observed in 8 femoral heads, including 3 in stage 2 (12.5%, 3/24) and 5 in stage 3–4 (100.0%). On tomographic images, increased ^18^F-FDG uptake was detected in 21 femoral head lesions, comprising 16 in stage 2 and 5 in stages 3–4.

**Fig. 1:**
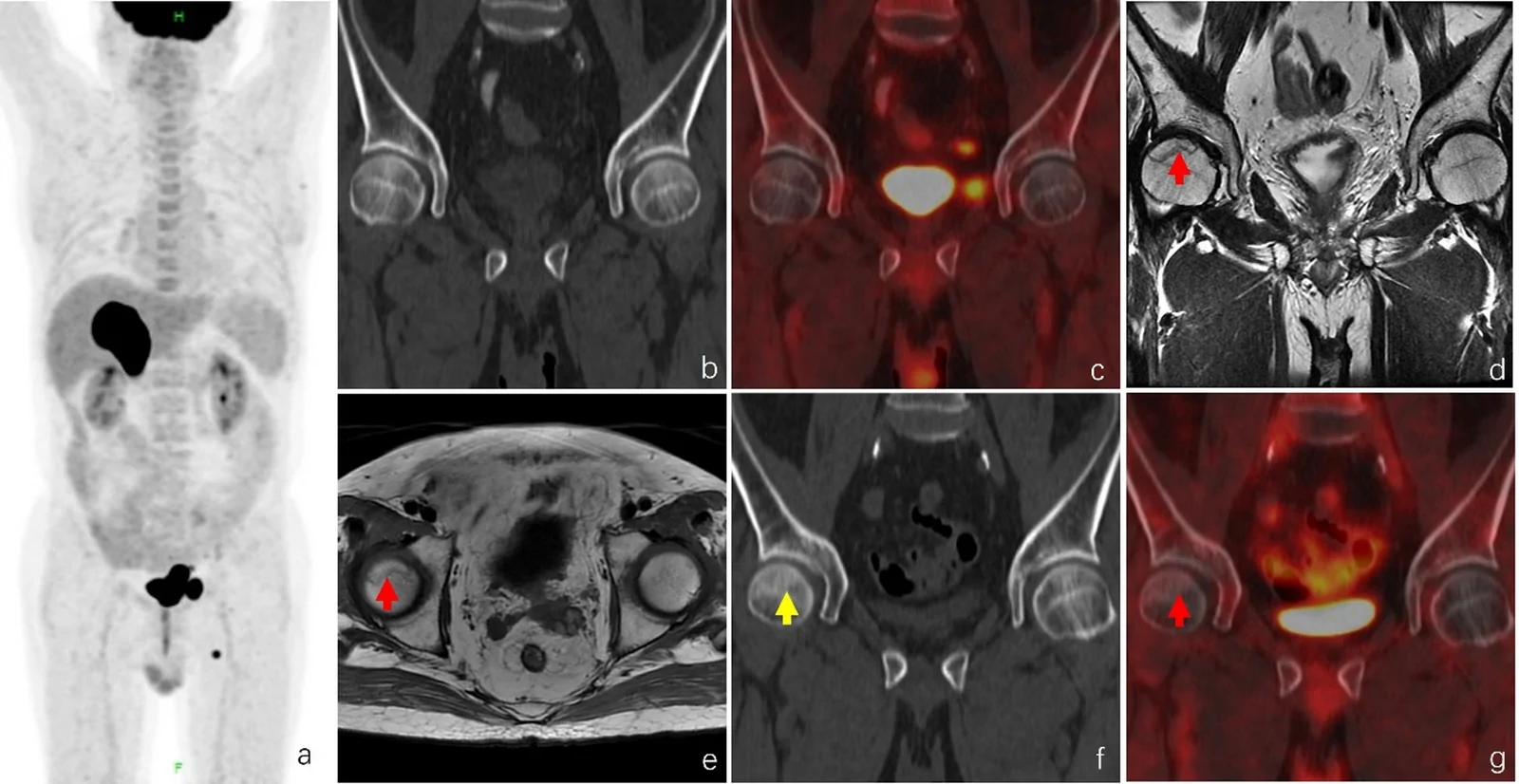
Osteonecrosis of the femoral head. PET/CT and MRI images from a 64-year-old male (No. 6) with diffuse large B-cell lymphoma after 4 cycles of chemotherapy. No abnormal findings were identified in the bilateral femoral heads on the maximum intensity projection (**a**), CT (**b**) and PET/CT fused image (**c**). However, an MRI scan performed on the same day as the PET/CT revealed an adipose-like high signal surrounded by linear hypointensity (arrow) at the right femoral head on the T2-weighted (**d**) and T1-weighted (**e**) images. According to the modified 2019 version of the Association Research Circulation Osseous international classification of osteonecrosis staging system, the osteonecrotic lesion of the right femoral head was classified as stage 1. A follow-up PET/CT was performed 46 months later and demonstrated a linear hyperdensity (arrows) at the right femoral head on CT (**f**) and PET/CT fused image (**g**)

**Fig. 2:**
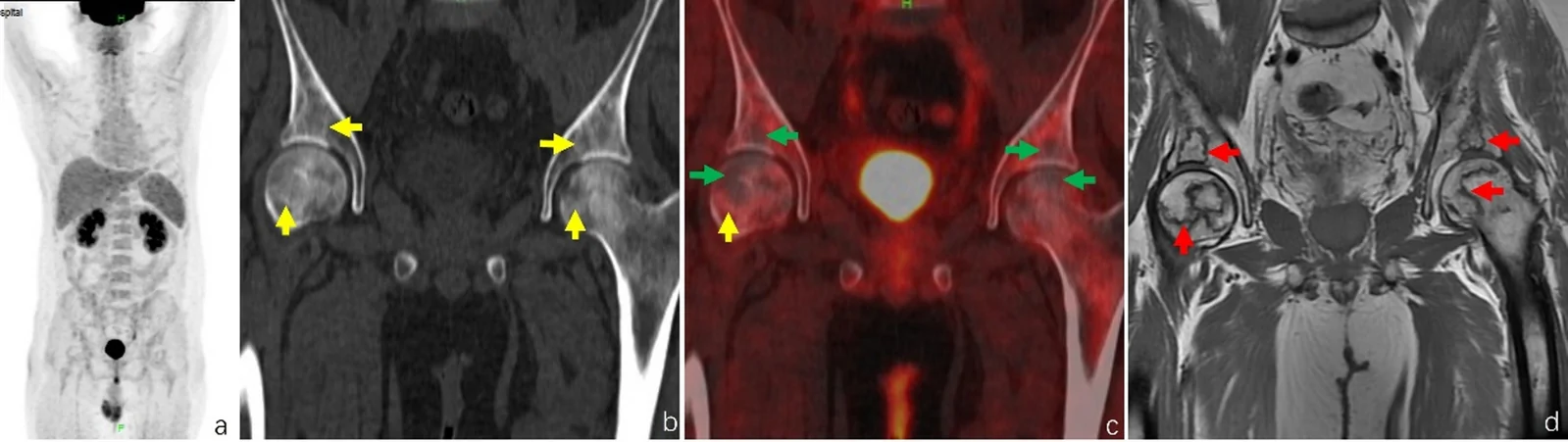
Osteonecrosis of the femoral heads and iliac bones. PET/CT and MRI images from a 25-year-old male (No. 7) with T-lymphoblastic lymphoma after allogeneic hematopoietic stem cell transplantation. The maximum intensity projection (**a**) and CT image (**b**) demonstrate multiple linear hyperdense lesions (yellow arrows) within the bilateral femoral heads and iliac bones. PET/CT fused image (**c**) reveals decreased ^18^F-FDG uptake (green arrows) with peripheral increased uptake (yellow arrow). An MRI scan performed the day after the PET/CT shows multiple geographic abnormal signals (red arrows) at the bilateral femoral heads and iliac bones on the T1-weighted image (**d**). According to the modified 2019 version of the Association Research Circulation Osseous international classification of osteonecrosis staging system, the osteonecrotic lesions of the bilateral femoral head are classified as stage 2

**Fig. 3:**
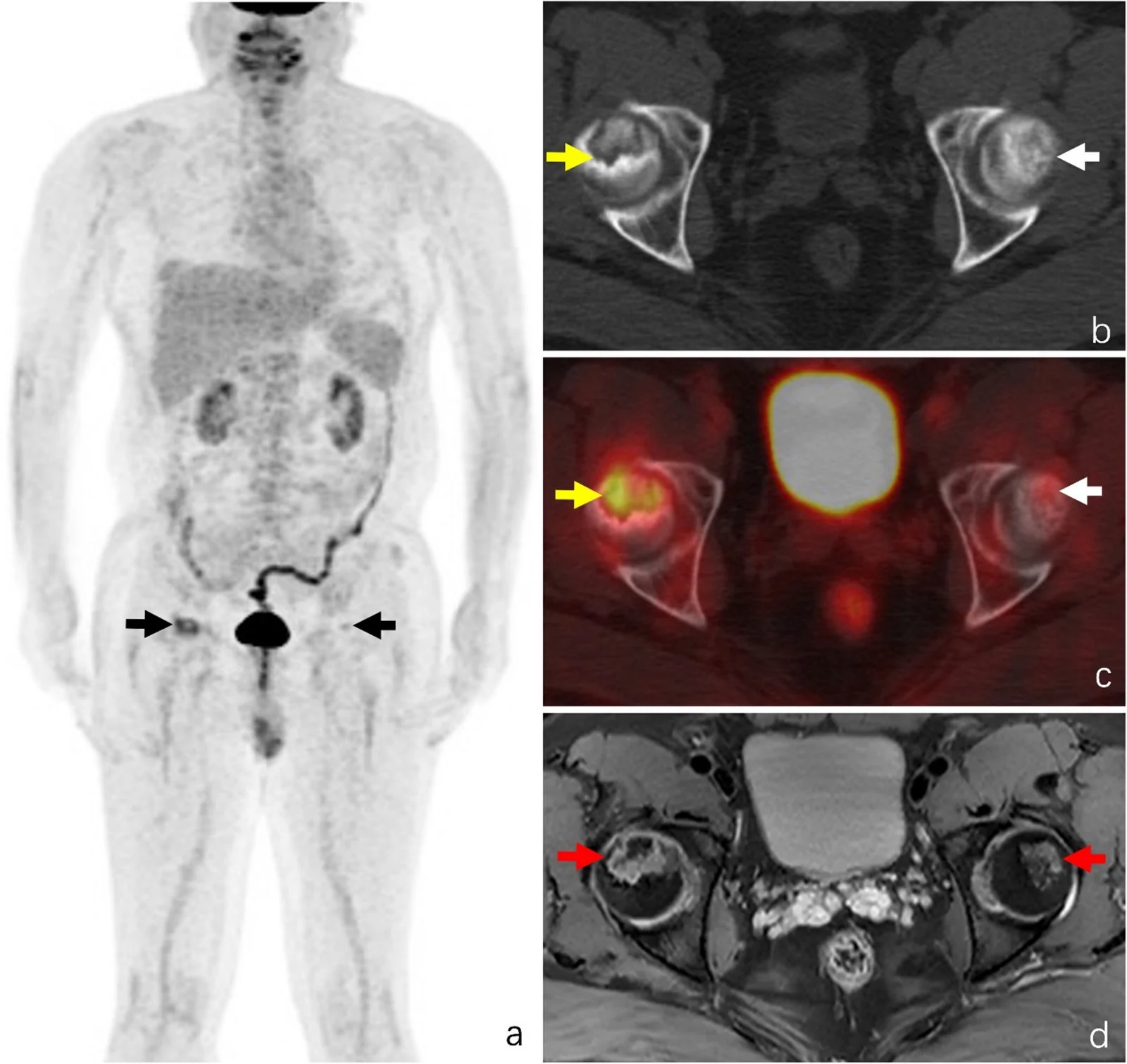
Osteonecrosis of the femoral heads. PET/CT and MRI images from a 44-year-old male (No. 11) with mantle cell lymphoma. The maximum intensity projection image of PET (**a**) shows increased ^18^F-FDG uptake (black arrows) in the regions of the bilateral femoral heads. The CT image (**b**) demonstrates osteolytic area with internal sequestrum, peripheral osteosclerosis, and subchondral fracture at the right femoral head (yellow arrow), as well as high density at the left femoral head (white arrow). The PET/CT fused image (**c**) shows increased ^18^F-FDG uptake at the bilateral femoral heads with SUV_max_ 4.41 (yellow arrow) and 2.23 (white arrow), respectively. An MRI scan performed three days after the PET/CT shows geographic abnormal signals at the bilateral femoral heads on the fat-saturated proton density-weighted image (red arrows in **d**). According to the modified 2019 version of the Association Research Circulation Osseous international classification of osteonecrosis staging system, the osteonecrosis in the right femoral head is classified as stage 3

In stage 1 femoral head lesions, both density and ^18^F-FDG uptake were normal (Fig. 1). For all stage 2 lesions, linear high density was observed at the edge of osteonecrosis on CT. Among these stage 2 lesions, 11 exhibited increased ^18^F-FDG uptake along the peripheral linear high-density and/or within the necrotic region, 5 showed peripheral increased uptake with internal decreased uptake (Fig. 2), 2 demonstrated decreased ^18^F-FDG uptake, and 6 had normal ^18^F-FDG uptake. All 5 lesions in stages 3 and 4 displayed increased ^18^F-FDG uptake along the peripheral linear high-density and/or at the osteolytic area (Fig. 3).

Regarding bone marrow background activity, three patients showed diffuse, relatively homogeneous increased ^18^F-FDG uptake in the bone marrow, suggestive of reactive changes. In one of these patients, this resulted in relatively decreased ^18^F-FDG uptake in the bilateral necrotic femoral head region compared to the surrounding reactive marrow (Supplementary Fig. 1). The remaining patients showed no increased bone marrow uptake. Importantly, no focal or pathological bone marrow activity due to active lymphoma or iatrogenic causes (e.g., colony-stimulating factors) was present that could have masked the ONFH lesions.

Among the 24 lesions in stage 2, 13 were confined above the epiphyseal line and 11 extended below the epiphyseal line on MRI. All 5 lesions in stages 3–4 extended below the epiphyseal line, and the extent of these lesions in stages 2–4 as seen on CT was consistent with that on MRI.

Twelve patients had previous PET/CT scans. On these previous PET/CT examinations, which were conducted a median of 27 months (range, 2–63 months) before MRI, linear high-densities were observed in the bilateral femoral heads of 11 patients, with 4 of them showing bilateral increased ^18^F-FDG uptake in the corresponding high-density areas. Five patients had follow-up PET/CT scans 4–46 months after MRI, involving a total of 8 femoral head lesions. One lesion progressed from stage 2 to stage 3. A stage 1 lesion exhibited a new linear high density (Fig. 1). The other 6 lesions showed no significant changes.

Nine patients exhibited osteonecrosis in bones other than the femoral head. MRI of the hips also revealed geographic abnormal signals in the bilateral iliac bones of 6 patients, characterized by peripheral low signal intensity and internal high signal intensity on T1-weighted images. On PET/CT, the iliac bone lesions showed peripheral linear high densities and internal decreased ^18^F-FDG uptake in 5 patients (Fig. 2), and isodensity with isometabolism in one patient. Regarding regions not covered by hip MRI, the humeral heads were affected in 9 patients (8 bilateral, 1 unilateral), showing linear high densities on CT, with 4 of them demonstrating increased ^18^F-FDG uptake along these linear high densities. Additionally, one patient had multiple involvements of the vertebrae, ribs, and scapulae.

## Discussion

This study demonstrated that ^18^F-FDG metabolism varies across different stages of ONFH. Specifically, increased ^18^F-FDG uptake was observed in 100.0% (5/5) of stage 3–4 lesions and 66.7% (16/24) of stage 2 ONFH. The median SUV_max_ of lesions in stage 3–4 was higher than that in stage 1–2. In the early stages of ONFH, the blood supply to the epiphysis is typically blocked, leading to trabecular bone and marrow necrosis. Theoretically, ^18^F-FDG uptake in the necrotic region should decrease. However, detecting this reduction can be challenging due to the low level of ^18^F-FDG metabolism in a normal adult femoral head [14]. Adult femoral heads are primarily composed of yellow bone marrow, which has limited vasculature and consists of approximately 95% adipocytes [15]. As the disease progresses, new bone forms around the necrotic area, appearing as linear high-density regions on CT and potentially increasing ^18^F-FDG uptake on PET. This characteristic was evident in 16 of 24 stage 2 lesions in this study. Subchondral fractures, granulation tissue, and osteoarthritis may contribute to the increased ^18^F-FDG uptake in stage 3–4 lesions. Furthermore, in patients with lymphoma, bone marrow ^18^F-FDG uptake is influenced by various factors, including tumour infiltration, anaemia, and colony-stimulating factors [16]. Against the backdrop of increased skeletal marrow metabolism, focal relatively decreased ^18^F-FDG uptake may be observed in the necrotic area, as was observed in one patient in this study (affecting both femoral heads). Importantly, femoral head involvement by lymphoma is rare and typically presents with markedly increased ^18^F-FDG uptake, with or without osteolytic destruction—features readily distinguishable from the osteonecrosis patterns seen in this study [17].

Karimova et al. [18] conducted a retrospective analysis of the clinical data and MRI images from 80 children and adolescents with haematological diseases and ONFH. They found that lesion size was the best prognostic factor for ONFH. Specifically, patients with lesions exceeding 30% of the femoral head were more likely to experience disease progression and require joint replacement surgery. In the present study, the epiphyseal line of the femoral head was used as a reference to assess the extent of the lesions. It was observed that all stage 3–4 ONFH lesions extended below the epiphyseal line, while 13/24 of stage 2 lesions were confined above the epiphyseal line. The proportion of large lesions (those extending beyond the epiphyseal line) was higher in stage 3–4 compared to stage 1–2. HU et al. [19] reported that for patients with stage 3 or higher ONFH, CT and MRI showed a high degree of consistency in accurately depicting the location, shape, and spatial structure of the lesion. In this study, the extent of ONFH lesions in stages 2–4 shown on CT was consistent with that on MRI, despite the relatively low-dose scan parameters used for the CT portion of PET/CT. Therefore, PET/CT may be a useful tool in assessing the extent of ONFH.

Osteonecrosis involving three or more separate anatomic sites of the skeleton concurrently or consecutively is defined as multifocal osteonecrosis [20]. Multifocal osteonecrosis has been reported in 3% to 11% of patients diagnosed with osteonecrosis [21, 22]. In this study, patients with bilateral ONFH accounted for 82.4% (14/17). Additionally, nine patients exhibited osteonecrosis in other bones, including the humeral heads (9 patients, 8 bilateral) and the bilateral iliac bones (6 patients). Consequently, more than half (9/17) of the patients had multifocal osteonecrosis. We speculate that lymphoma-associated ONFH may represent a localized manifestation of systemic osteonecrosis [20, 23, 24]. Therefore, it is crucial to evaluate the entire skeleton in patients with lymphoma-associated ONFH. Although MRI is considered the optimal method for diagnosing osteonecrosis, screening for multifocal osteonecrosis using MRI can be time-consuming and costly. Bone scintigraphy may offer a useful alternative for diagnosing multifocal osteonecrosis [21, 22]. The present study suggests that PET/CT may play a role in detecting multiple skeletal lesions in patients with lymphoma-associated ONFH.

In 11 patients, PET/CT scans conducted a median of 27 months (range 2–63 months) prior to the MRI diagnosis of ONFH revealed abnormal imaging features in the femoral heads. Regrettably, these abnormal features were overlooked in the initial PET/CT interpretation for 9 of these patients. Similar findings were reported by Barille et al. [25], who noted that the diagnosis of femoral head avascular necrosis was frequently missed (89%) on pelvic CT performed for alternative primary indications. Therefore, when interpreting PET/CT scans for lymphoma evaluation, it is crucial to routinely assess for signs of osteonecrosis to prevent delays in diagnosis and treatment. Lesions with low ^18^F-FDG uptake may be easily missed on PET scans. In this study, while only 12.5% (3/24) of stage 2 ONFH lesions showed increased uptake on the MIP images, a significantly higher proportion, 66.7% (16/24), demonstrated increased uptake on the tomographic images. Consequently, greater emphasis should be placed on examining the tomographic PET and CT images to identify features of ONFH.

The present study had several limitations. Firstly, the retrospective nature of the study and the relatively small sample size may have influenced the results. The study design was a descriptive case series without a control group, precluding assessment of diagnostic performance metrics such as sensitivity, specificity, or predictive values. Secondly, the diagnosis of ONFH was based solely on MRI findings and lacked histological confirmation. The readers were not blinded to MRI results, which could introduce bias. Additionally, we did not establish a specific threshold for SUV_max_ in diagnosing ONFH, due to the variable degree of ^18^F-FDG uptake observed in the lesions. The long time interval between some PET/CT and MRI examinations (up to 98 days) may weaken lesion-level correlation. Given these considerations, we recommend that greater attention be given to CT features when raising suspicion for ONFH, as they can provide valuable supplementary information. Future studies with larger sample sizes, a control group, blinded readers, and standardized MRI protocols are needed to validate our findings and assess the diagnostic accuracy of PET/CT for ONFH.

## Conclusions

In patients with lymphoma, osteonecrosis of the femoral head exhibits variable degrees of ^18^F-FDG uptake, which is related to different pathological stages. Additionally, it may be accompanied by involvement of other bones, such as the humeral heads and iliac bones. PET/CT may raise suspicion for ONFH and detect multiple bone involvements during lymphoma assessment; however, its diagnostic accuracy requires prospective validation against standardized MRI.

## Electronic Supplementary Material

Below is the link to the electronic supplementary material.

**Supplementary Material 1: Supplementary Fig. 1.** Osteonecrosis of the femoral heads. PET/CT and MRI images from a patient (No. 16) with T-cell lymphoblastic lymphoma. The maximum intensity projection (a) shows multiple hypermetabolic lymph nodes in the cervical, supraclavicular, mediastinal, abdominal, and retroperitoneal regions; splenomegaly with increased uptake; and diffusely increased bone marrow metabolism. The CT image (b) demonstrates linear hyperdense lesions (arrows) at the bilateral femoral heads, with decreased uptake inside the hyperdense rim (arrows) on the PET/CT fused image (c). An MRI scan performed two days after the PET/CT revealed an adipose-like high signal surrounded by linear hypointensity (arrows) at the bilateral femoral heads on the T1-weighted image (d)

## Data Availability

The analysed and/or used datasets in this study can be obtained on reasonable request to the corresponding author.

## Abbreviations

FDG: Fluorodeoxyglucose
MIP: Maximum intensity projection
ONFH: Osteonecrosis of the femoral head
SUV_max_: Maximum Standardized uptake value
